# Biochar, microbes, and biochar-microbe synergistic treatment of chlorinated hydrocarbons in groundwater: a review

**DOI:** 10.3389/fmicb.2024.1443682

**Published:** 2024-07-18

**Authors:** Shixin Niu, Changsuo Li, Shuai Gao, Jingya Tian, Chao Zhang, Lixia Li, Yao Huang, Honghong Lyu

**Affiliations:** ^1^Shandong Provincial Geo-mineral Engineering Exploration Institute, Shandong Provincial Bureau of Geology & Mineral Resources, Jinan, China; ^2^Shandong Engineering Research Center for Environmental Protection and Remediation on Groundwater, Jinan, China; ^3^Tianjin Key Laboratory of Clean Energy and Pollution Control, School of Energy and Environmental Engineering, Hebei University of Technology, Tianjin, China; ^4^Guangdong Key Laboratory of Integrated Agro-Environmental Pollution Control and Management, Institute of Eco-Environmental and Soil Sciences, National-Regional Joint Engineering Research Center for Soil Pollution Control and Remediation in South China, Guangdong Academy of Sciences, Guangzhou, China

**Keywords:** biochar, CHCs, dehalogenation respiratory anaerobic bacteria, dechlorination, enhancement

## Abstract

Dehalogenating bacteria are still deficient when targeted to deal with chlorinated hydrocarbons (CHCs) contamination: e.g., slow metabolic rates, limited substrate range, formation of toxic intermediates. To enhance its dechlorination capacity, biochar and its composites with appropriate surface activity and biocompatibility are selected for coupled dechlorination. Because of its special surface physical and chemical properties, it promotes biofilm formation by dehalogenating bacteria on its surface and improves the living environment for dehalogenating bacteria. Next, biochar and its composites provide active sites for the removal of CHCs through adsorption, activation and catalysis. These sites can be specific metal centers, functional groups or structural defects. Under microbial mediation, these sites can undergo activation and catalytic cycles, thereby increasing dechlorination efficiency. However, there is a lack of systematic understanding of the mechanisms of dechlorination in biogenic and abiogenic systems based on biochar. Therefore, this article comprehensively summarizes the recent research progress of biochar and its composites as a “Taiwan balm” for the degradation of CHCs in terms of adsorption, catalysis, improvement of microbial community structure and promotion of degradation and metabolism of CHCs. The removal efficiency, influencing factors and reaction mechanism of the degraded CHCs were also discussed. The following conclusions were drawn, in the pure biochar system, the CHCs are fixed to its surface by adsorption through chemical bonds on its surface; the biochar composite material relies on persistent free radicals and electron shuttle mechanisms to react with CHCs, disrupting their molecular structure and reducing them; biochar-coupled microorganisms reduce CHCs primarily by forming an “electron shuttle bridge” between biological and non-biological organisms. Finally, the experimental directions to be carried out in the future are suggested to explore the optimal solution to improve the treatment efficiency of CHCs in water.

## 1 Introduction

**Figure 1:**
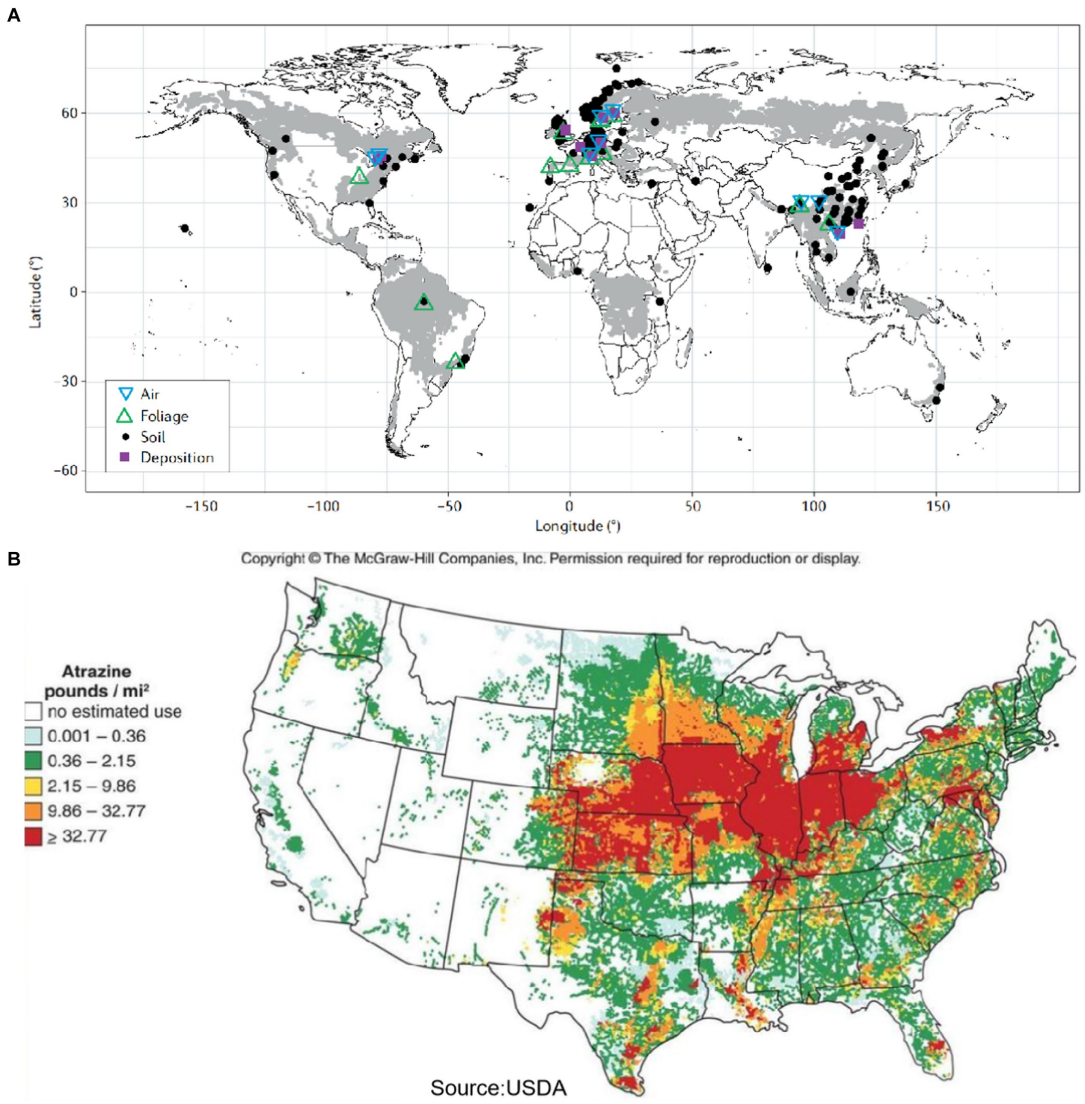
**(A)** Global distribution of persistent organic pollutants pollution (Gong et al., 2021) and **(B)** map of CHCs usage in US (only available for US as per the literature) (Greene, 2019).

Chlorinated hydrocarbons (CHCs) are compounds containing chlorine, carbon, and hydrogen. The term encompasses organochlorine pesticides such as lindane and dichlorodiphenyltrichloroethane, industrial chemicals like polychlorinated biphenyls (PCBs), trichloroethylene (TCE), 1,2,4-trichlorobenzene (1,2,4-TCB), and chlorine wastes such as dioxins and furans. These compounds are important organic chemicals widely used as industrial raw material and organic solvent. There are currently significant challenges in remediating CHCs pollution. As shown in Figure 1A, persistent organic pollutants are predominantly concentrated in mountainous forest regions worldwide, with smaller amounts detected in developed areas. Figure 1B provides a detailed map of CHCs usage in the United States, due to improper use and handling, such as industrial wastewater discharge, leakage during production and transport, and volatilization in the environment, result in serious contamination of soil and groundwater. CHCs are particularly problematic due to their high chemical stability and low solubility, which cause them to accumulate at the bottom of aquifers, forming dense non-aqueous phase liquids with densities greater than that of water. This accumulation leads to expanding areas of contamination, forming persistent sources of pollution that pose environmental and public health risks (Wu et al., 2021). Additionally, the complexity of polluted environments, such as groundwater and soil heterogeneity, changes in mobility, and varying strata, increases the uncertainty of contaminant migration and distribution, further complicating remediation efforts. Research into technologies for the complete dechlorination of CHCs in groundwater is essential.

In this context, biochar (BC) and its composites, combined with microbial remediation technology, have proven valuable. As a green and low-cost catalyst material, biochar and its composites are widely used due to their strong adsorption structure and surface-active groups. Studies have shown that biochar (SB) produced by pyrolysis of sawdust can adsorb ciprofloxacin (CFX) and sulfamethoxazole (SMX), with maximum adsorption capacities of 3.54 and 3.40 mg/g, respectively (initial concentration 20 mg/L, removal rates above 85%). Kinetic fitting indicated that the adsorption of both compounds follows pseudo-second-order kinetic behavior (Silori et al., 2023). After capturing CHCs molecules, surface active substances [such as carboxyl groups -COOH, hydroxyl groups -OH, metal atoms (Cheng et al., 2024), non-metallic active substances N and S, etc. (Yang et al., 2020)] and/or electron transfer react with the CHCs molecules on the surface, rendering them harmless and reusable. Some studies have selected mugwort (low ash and equivalent nitrogen content) to produce biochar and catalyze the sulfide reduction of perchloroethylene under alkaline conditions. These studies have shown that -COOH and pyridinic nitrogen are key in reducing perchloroethylene. Specifically, after deprotonation of -COOH, the adjacent carbon atoms become electron-deficient sites, while the carbon atoms attached to pyridinic nitrogen form Lewis base sites. These sites bind electrostatically with reducing sulfides, forming catalytic sites that attract the reduction of perchloroethylene to produce acetylene (Yang et al., 2022). Furthermore, bioremediation is another promising approach for *in situ* remediation of subsurface contamination. During bioremediation, dehalogenating respiratory anaerobic bacteria in underground aquifers use CHCs as electron acceptors for metabolism. This process gradually reduces or replaces chlorine atoms, thereby maintaining the environmental background concentration. Different types of dehalogenating respiratory anaerobic bacteria can have synergistic effects in the degradation activities and are responsible for reactions in different steps, improving the overall degradation efficiency (Wang et al., 2019). For instance, *Dehalococcoides mccartyi 195* has an incomplete acetyl-CoA pathway, necessitating the cleavage of acetyl-CoA to produce methyltetrahydrofolate for methionine biosynthesis when using formate, with CO as a mandatory by-product. Accumulation of CO inhibits the metabolic growth and dechlorination function of *195* (Zhuang et al., 2014). This issue can be mitigated by coexisting with CO-metabolizing anaerobic bacteria like *Octococcus methanogenes*, which feeds on CO and produces acetate and formate. Thus, *195* and *Octococcus methanogenes* form a mutually beneficial symbiotic partnership that enhances the dechlorination environment for *195* (Rother and Metcalf, 2004). Recent research on biochar, its composites, and dehalogenating respiratory anaerobic bacteria for treating CHCs in groundwater has deepened. Biochar regulates the structure and function of microbial communities, promoting the growth and activity of specific bacterial strains. Additionally, biochar provides active oxidants or reductants, improves electron utilization, and increases the efficiency of dechlorination reactions (Li et al., 2023).

Groundwater dechlorination technology has garnered significant attention since CHCs contamination was identified. This paper discusses the synthesis of biochar and its composites, dehalogenating respiratory anaerobes and their synergistic removal of CHCs from groundwater, focusing on removal efficiencies, influencing factors and removal mechanisms. In addition, future research directions are proposed to provide a theoretical basis for the remediation of CHCs in groundwater and risk management.

## 2 Removal of CHCs by biochar and its composites

### 2.1 Introduction of biochar and its composites

The resulting material is characterized by low production costs, a wide range of raw materials and a simple preparation process. It serves as a new material with both adsorption and catalytic functions. Biochar can be viewed as a continuum of complex carbonaceous materials such as cellulose, carboxylic acids and their derivatives, phenols, paraffins and olefin derivatives (Li et al., 2011). Carbonaceous materials produced by biomass carbonization have electron transport properties (related to microporous structure, carbon defects and condensed aromatic groups) and participate in biogeochemical redox reactions (Liu et al., 2024).

However, biochar has shortcomings such as small particle size, light weight and easy agglomeration. These problems can be effectively addressed by loading metal oxides, bimetallic materials, nanomaterials or microorganisms onto the surface of biochar (Regmi et al., 2012). The preparation methods of composites mainly include chemical co-precipitation method, high-energy ball milling method, pyrolysis method and immersion precipitation method (Huang et al., 2019). The performance improvement of biochar composites is mainly due to the interaction between biochar and the load materials. Biochar, as a carrier, can also enhance the activity of the loading material and increase the contact probability between the adsorption sites and CHCs. The loading process can also affect the pore structure of the biochar itself, such as pore volume and pore size. For instance, biochar produced from waste palm husks (BET = 185.8 m^2^/g) was used for water dechlorination. Combined microwave heating and physicochemical modification resulted in a higher surface area (717.8 m^2^/g) and a higher adsorption capacity (35.8 mg/g) compared to single modifications with steam (527.4 m^2^/g; 25 mg/g) or KOH (301.1 m^2^/g; 23 mg/g) alone (Shi et al., 2022). The addition of metal oxides and non-metallic element sites to the surface of biochar can increase the functional groups on the surface of biochar. While removing CHCs by physical adsorption, biochar chemically reacts with CHCs to enhance the degradation effect. For example, nitrogen-doped biochar prepared from alfalfa was used to catalytically degrade carbon tetrachloride (CT) in an aqueous solution containing sulfur compounds. The main dechlorination products are Cl^−^, chloroform (*CF*), CS_2_ and HCO_3_^−^. The catalytic hydrogenolysis of CT to *CF* was found to be due to enhanced electron transfer of N5, GN and quinone/hydroquinone. The formation of CS_2_ is attributed to the interaction between N6 and sulfide. This study clarified that the means of catalytic CT dechlorination include catalytic nucleophilic substitution of SN_2_ (main means) and catalytic hydrogenolysis (auxiliary means) (Ding et al., 2022). Another example involves peanut shells produced by the carbonization-hydrothermal-chemical reduction method to produce palladium-loaded magnetic biochar. Electrochemical methods were used to catalyze the dechlorination of pentachlorophenol. After 10 dechlorination cycles, the removal efficiency decreased by only 0.2% (99 to 98.8%), and the dechlorination efficiency could still reach 90.1%, reflecting good stability and recyclability (Wang Y. et al., 2021).

### 2.2 Biochar and its composites remediate CHCs contamination in groundwater

There are significant differences in the remediation efficiencies of biochar and its composite materials for CHCs in water under different treatment conditions. Factors such as different biochar feedstocks, biochar pyrolysis temperatures and material compositing methods all affect the performance of the final treated materials.

#### 2.2.1 The effect of biochar feedstocks on the removal efficiency of CHCs

The physical and chemical properties of biochars produced from different feedstocks during the pyrolysis process have a direct impact on their degradation of CHCs. Generally, biochar produced from aromatic plant feedstocks has a large specific surface area, rich pore structure and strong electronic storage capacity (ESC), which not only effectively absorb CHCs but also provides a growth substrate for microorganisms attached to its surface, forming an electron transport chain. This not only helps to capture CHCs from the environment on the biochar surface, but also provides a good starting environment for subsequent degradation (Xin et al., 2023). Components such as ash in the feedstock in the feedstock can also affect the removal of CHCs (Schreiter et al., 2018). Commonly used biochar feedstocks include wood chips, biogas residue, sewage sludge. Feedstocks with high lignin content can increase the production capacity of biochar and modified biochar. Compared to wood biochar, biochars from sewage sludge, animal manure, and digestate contain large amounts of organic and inorganic matter, low carbon content, high ash content, and may contain heavy metals and other contaminants. Pyrolysis-produced biochar is mostly mesoporous and macroporous, with an uneven pore size distribution that can be easily blocked by ash and inorganic matter, resulting in poor conductivity. This interferes with the growth and metabolism of coupled dehalogenating bacteria, making it difficult to establish an “electron shuttle bridge” with dehalogenating bacteria to reduce CHCs (Qiu et al., 2021). Under the same conditions (pyrolysis at 550°C for 2 h), the electron transfer capacity of straw biochar (0.89 mmol/g) is lower than that of pig manure biochar (2.18 mmol/g), but straw biochar (260.21 m^2^/g) has a significantly larger than pig manure biochar (167.88 m^2^/g). This suggests that at this pyrolysis temperature, the manure biochar retains more oxygen-containing functional groups that form electron shuttle tunnels and can easily transfer electrons to CHCs and promote their reductive dechlorination (Li L.-P. et al., 2022). The modified biochar has a large specific surface area and a developed pore structure, which is thought to be due to the formation of a support structure for the modified target substance between the layers – ‘pillars’ – which helps to form a microporous structure. At the same time, the outer surface of the biochar is layered with nanoparticles with sizes larger than the diameter of the mesopores and micropores, contributing to the increase in specific surface area (Ahmad et al., 2022). There are currently many studies on the effect of biochar feedstocks on biochar performance. The following Table 1 lists their effects on dechlorination performance in recent years.

**Table 1:** The effect of different precursors, pyrolysis temperatures and their modification on the reductive dechlorination of the biochar produced.

Raw materials	Pyrolysis temperature (°C)	Modification method	Bio-charcoal precursor	Specific Surface area (m^2^/g)	Active substance	CHCs	Dechlorination effect	Dechlorination products	Reference
nZVI-BC	500, 600, 700	Chemical reduction	Cornstalks and FeCl_3_	28.5 31.2 64.8	-OH, C=C, C=O, C-O, Fe	TCE 30 ppm	Within 4 h >85%	Ethylene	Dong et al. (2017)
SBC/nZVI-Pd	300, 500, 700	Chemical reduction, NaOH activation	Straw and FeSO_4_	2.98 167.57 262.19	C=C, C=O, C-O, Fe/O	1,2,4-TCB 160 ppm	90.2% 80.6% 98.8%	Benzene	Shang et al. (2020)
Ni/Fe-BC	500, 600	Liquid phase reduction	Wheat Straw, FeSO_4,_ NiSO_4_	–	Atomic Hydrogen	1,1,1-Trichloroethane 20 ppm	99.3%	Ethane	Li et al. (2017)
Mba	600	Pyrolysis	Maple	613.6	C=O, C=C, Laccase	Dichlorobiphenyl 40 ppm	71.4%	–	Li et al. (2018)
nZVI-CBC	–	Liquid phase reduction	–	872.5	Atomic Hydrogen	TCE 25 ppm	65%	Ethane	Hou et al. (2024)
Ni/Fe-CS	700	Chemical reduction	Corn stalks	60.1	Atomic Hydrogen	Trichlorobenzene 154.14 μmol/L	93%	Benzene	Han et al. (2019)
N-BC	600, 700, 800	HNO_3_	Alfalfa	330, 449, 755	N5, N6	Carbon Tetrachloride 0.4 mmol/L	75%	Cl^−^, *CF,* CS_2,_ HCO_3_^−^	Ding et al. (2022)
Bc-Fenton-RM	800	Pyrolysis	Sludge	51.15	Fe-O, ·OH	Chlorophenol 2.34 mmol/L	100%	–	Gan et al. (2020)
BC-CM BC-GH BC-WC	450	–	Cow dung (CM) Chaff (GH) wooden boat (WC)	6 215 341	Pore filling	TCE, tetrachlorethylene	BC-CM < BC-GH < BC-WC	–	Schreiter et al. (2018)
SDBC	400	NaOH or HNO_3_	Sludge	3.85, 10.12	·OH	*p*-chlorophenol 5 ppm	90%	CO_2_、H_2_O	Jiang et al. (2022)
PSBC	600, 1,000	Persulfate Activation	Pharmaceutical Sludge	78.4, 59.0	SO_4_^·−^ ·OH, FeNx, FeIVO_2_+	Tetrachlorophenol 0.1 mmol/L	20 min, 100%	Propionic acid, acetic acid	Liang et al. (2023)
Ni-ZVI-MBC	700	Ball milling	Paper mill sludge	167.86	Atomic Hydrogen	Pentachlorophenol 10 ppm	60 min, 90%	Benzene	Devi and Saroha (2015)
BC-Fe	400	Chemical reduction	Sludge	-	-OH, -C=O, COO-, Fe-O	Dicamba 20 ppm	180 min, 92%	3,6-Dichlorosalicylic acid 3,6-Dichlorogentisic acid	Wan et al. (2021)
Fe^0^ -BRtP	228	Chemical Reduction	Rambutan peel	109.57	-OH, -C=O, Fe-O	Various organochlorine pesticides 2 ppm	20 min, 66%	Dichlorobenzophenone, 1,2-dichlorobenzene	Batool et al. (2022)
HPMC/PS/BC	550	Persulfate	Bamboo	–	SO4^·−^, ·OH	TCE 10 ppm	100%	CO_2_, H_2_O	Nguyen et al. (2023)

#### 2.2.2 The effect of biochar pyrolysis temperature on the removal of CHCs

Pyrolysis temperature (PT) affects the dechlorination performance of biochar. Specifically, pyrolysis at lower PT (around 300°C) mainly involves the volatilization of organic matter in the biochar and the dissociation of lighter carbon chains. With an increase of PT (above 600°C), the carbon chain further decomposed, and the organic material gradually transformed into a structure containing an aromatic carbon skeleton, with a higher degree of carbonization, reduced surface functional groups, weakened polarity, and enhanced electrical conductivity of the biochar (Tan et al., 2016; Lyu et al., 2020). Some studies have found that an ordered graphite layer structure can reduce TCE through rapid electron transfer in the electron conduction domain (Xu et al., 2013). Cornst over was used as a feedstock for biochar production (Meng et al., 2018). It was found that the elemental composition of biochar changed greatly with pyrolysis temperature, and the C content increased from 70.34% (BC-250) to 88.62% (BC-850) with increasing PT. The content of N, H and O elements decreases, and the surface functional groups decrease. Biochar prepared at 550°C has the highest adsorption capacity of trichlorethylene, with a maximum adsorption capacity of 984.9 mg/g, while biochar prepared at 850°C has a maximum adsorption capacity of only 346.9 mg/g. This is likely because, when the PT exceeds a certain threshold, the carbon structure of the biochar is lost, causing some of the active factors beneficial to dechlorination (such as conductivity, aromaticity, surface functional groups, etc.) to disappear. Studies have shown that when biochar is used as an electron transfer mediator and used of sheet Fe(II)–Fe(III) hydroxide (green rust) as reducing agent, the dechlorination (TCE) activity increases with PT (450 to 1,050°C). The most significant quasi-primary kinetic constant of 2.0 h^−1^ was obtained under the biochar pyrolysed at 950°C, while this phenomenon was not observed at lower temperatures (450°C) (Ai et al., 2020). The reason is that the reactivity is closely related to the electron acceptance capacity (EAC) of BC.

#### 2.2.3 The effect of preparation method of composites on the removal efficiency of CHCs

Different methods of compositing produce carbon materials with different properties. Commonly used methods for producing biochar-based composites include chemical co-precipitation, impregnation precipitation, high-energy ball milling and pyrolysis (Huang et al., 2019). The chemical co-precipitation method is simple to prepare, the experimental conditions are easy to control and the cost is low, but the added precipitant can lead to excessive local concentration, causing agglomeration or insufficiently homogeneous composition, and the bonding stability of the composites produced is poor. The precipitation process can be optimized by controlling pH and temperature. For example, nMnOx@Biochar was prepared by the chemical precipitation method and used to activate persulfate (PMS) for chlorophenol removal. The results showed that under 0.2 mmol PMS, the degradation rate of chlorophenol within 60 min is 71%, and the final products of dechlorination are CO_2_ and H_2_O (Liu T. et al., 2021). The components impregnated by the impregnation-precipitation method can be uniformly distributed on the surface of the support, but the operation is complicated and subsequent processing may affect the performance of the material particles. It can be adjusted by controlling the concentration of materials in the impregnation solution and the impregnation time. The pyrolysis method has fewer preparation steps, the conditions are easy to control and the combination of feedstock and biochar is relatively stable. The pyrolysis method was used to uniformly load iron ions on biochar *in situ* to produce nZVI-BC for adsorption and removal of polychlorinated biphenyls. The study found that nZVI-BC had the best removal efficiency for trichlorobiphenyl (6d, 86%). This is mainly due to the good graphitised structure of BC and the loading of nZVI enriches the pore structure where electrons involved in redox can be efficiently transported to achieve PCB removal (Liu et al., 2016). High-energy ball milling is one of the most important methods for producing new high-performance materials. It is not only easy to operate and inexpensive, but can also increase powder activity, improve particle distribution and enhance the bonding ability between matrices. Therefore, many studies have used ball milling to prepare composite materials. Ball milling technology was used to load FeS on nitrogen-doped biochar and phenol was degraded by persulfate activation. The results show that Fe, N and S elements are uniformly distributed on the surface of biochar and form a developed pore structure. The degradation rate of phenol reached 76% within 60 min, and the final mineralization products were CO_2_ and H_2_O (Qu et al., 2022). In addition to the above methods for preparing biochar composites, active substance can also be injected directly into the reaction system and biochar can be used as an electron mediator (electron donor or electron acceptor). Specifically, by using layered iron(II)–iron(III) hydroxides [green rusts (GRs)] as a reducing agent for methane chloride, biochar acts as an electron mediator, achieving 85% removal of *cis*-dichloroethylene, *trans*-dichloroethylene and perchloroethylene within 24 h, and its main product is acetylene (>85%) (Ai et al., 2019).

The catalytic TCE reduction of biochar and its composites is considered to be a complex reaction process. The three influencing factors discussed above do not act in isolation. Different feedstocks, different pyrolysis temperatures during preparation, and different loading methods will all change. Biochar surfactants. TCE is adsorbed to the surface of biochar particles through hydrophobic interactions and then interacts with biochar particles through polar functional groups, as well as through redox-active substances and electron-mediated electron transfer-conducting domains in biochar to achieve degradation.

### 2.3 Reaction mechanism

Biochar primarily relies on physical and chemical processes to remove CHCs. The microscopic pore structure of biochar is key to its physical adsorption capacity, providing numerous adsorption sites that allow biochar to effectively adsorb CHCs. However, physical adsorption is dominated by van der Waals forces, which cause non-covalent interactions between the CHCs and the biochar surface. Since van der Waals forces are short-range and do not involve the formation of chemical bonds, this type of adsorption is reversible and can be released under appropriate conditions. The chemical process involves the formation of chemical bonds between biochar surface functional groups and CHCs, a process known as ‘CHCs detoxification.’ Specifically, the hydroxyl groups on the biochar surface form hydrogen bonds with chlorine atoms, the carboxyl groups form ionic bonds with the positive charges in the CHCs, and the ketone groups can bind the CHCs through covalent bonds. Additionally, π–π stacking occurs in the aromatic ring structure of biochar and the electron cloud density of CHCs.

**Figure 2:**
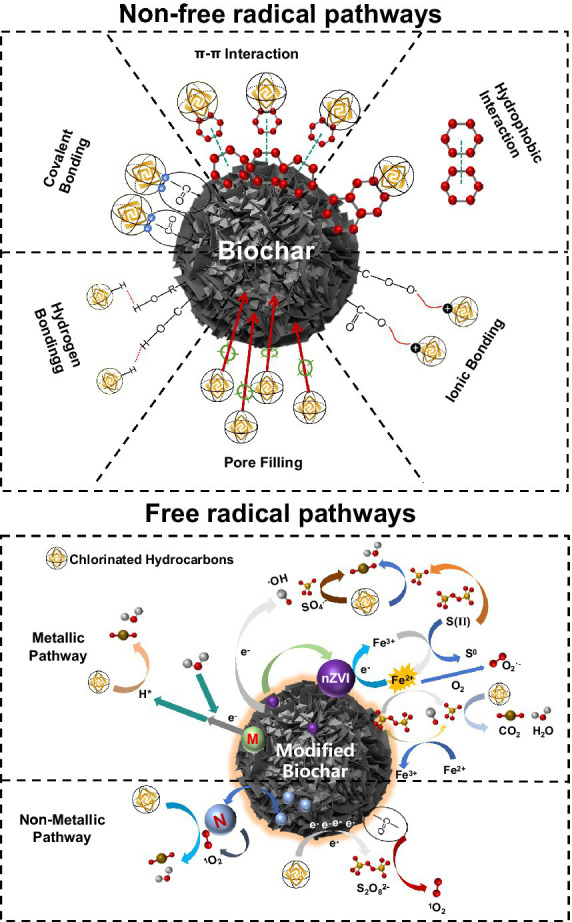
Mechanism diagram of the degradation of CHCs by biochar and biochar composite materials.

As shown in Figure 2, biochar can be doped with non-metallic elements (e.g., N, S, P, etc.), metallic elements (e.g., nickel, iron, manganese, etc.) and/or incorporated into active systems (e.g., persulfuric acid system) to enhance its inherent properties (adsorption, redox, biocompatibility, etc.). Nitrogen doping can introduce amine (-NH_2_) functional group and pyridine nitrogen group. The former enhances the surface polarity and hydrophilicity of biochar, improving the adsorption capacity of organic pollutants, similar to the effect of hydroxyl (-OH); the latter mainly enhances the catalytic activity of biochar. Sulfur doping can introduce thiol (-SH) functional groups, which mainly improve the reducing properties of biochar; phosphorus doping can introduce phosphate (-PO_4_) functional groups, which improve the ability of biochar to adsorb and degrade heavy metal ions. When the degradation process occurs in an active system (such as PMS), the presence of numerous active substances allow free radicals to be generated by the action of functional groups on the biochar surface (e.g., hydroxyl radical ·OH, superoxide radical O_2_^·-^, sulfate free radicals SO_4_^·-^, etc.). Doping with metallic elements promote the redox reaction, oxidizing some of the carbon atoms on the surface of the biochar to hydroxyl (-OH) and carboxyl (-COOH). In aqueous environments or soils with high water content, atomic hydrogen H* is also produced, enhancing the oxidative properties of the oxygen-containing functional groups on the surface of the biochar. While metallic elements prepare the charge, non-metallic elements (N, S, etc.) can be introduced to further enhance the activity of the reaction system.

The innovative design of biochar composites enhances adsorption and redox properties compared to biochar alone. Biochar composites provide intermediates or active centers (oxygen functional groups, persistent radicals, etc.) necessary for catalytic reactions on the excellent matrix of biochar (e.g., large specific surface area, excellent electrochemical activity, polarity, etc.). These composites not only show excellent removal performance for CHCs but also provide a solid foundation for coupling with dehalogenating bacteria, offering an excellent habitat.

## 3 Removal of CHCs by dehalogenation respiratory anaerobic bacteria system

### 3.1 Introduction of dehalogenation respiratory bacteria

Dehalogenating respiratory anaerobes usually belong to the group of sulfate-reducing or nitrate-reducing bacteria, etc., which have a low dependence on oxygen and are able to survive and multiply in anoxic and chlorine-rich environments such as groundwater, soils and sediments. Common dehalogenating respiratory bacteria include *Geobacter*, *Desulfomonas*, *Desulfovibrio*, *Sulfomonas*, *Desulfobacterium*, *Dehalobacter,* and *Dehalococcus* spp. etc. (Nijenhuis and Kuntze, 2016). Organohalogen-respiring bacteria play an important role in the removal of organochlorine compounds in anaerobic environments such as groundwater and sediments. For example, *Bacteroides dehalogenans* can dechlorinate pollutants like TCE and monochloroethylene into non-toxic ethylene (Löffler et al., 2013). Inoculants developed with *Bacteroidetes dehalogenans* as the core strain have many application examples for remediation of organochlorine pollution in groundwater and have ideal degradation effects on chlorinated alkenes and chlorinated alkanes, indicating that organohalogen-respiring bacteria play an important role at contaminated sites. Remediation value and potential (Paul et al., 2013). Some studies have used a lactic acid flow tower (as electron donor for *Bacteroidetes*) and a FeOOH packed tower (as electron acceptor for *Bacteroidetes*) in series to completely degrade 50 μM pentachlorophenol under 4 mM lactic acid and anaerobic conditions (carbon balance shows CO_2_ 81%, CH_4_ 3, 8% for microbial growth) and the mineralization rate reached 3.5 μM/d (Li et al., 2010). *Dehalomonas* is a new type of specialized organohalogen respiratory bacterium discovered in recent years, which belongs to the strictly anaerobic specialized organohalogen respiratory bacteria, which cannot use glucose, lactic acid or yeast paste and other substrates for anaerobic fermentation, and grows relatively slowly, with a cell doubling cycle of about 2d (Lu et al., 2021). Anaerobic enrichment cultures GP established by inoculation of grape pomace compost by some researchers were able to dechlorinate growth and complete dechlorination to ethylene using a variety of vinyl chloride analogs (e.g., TCE, 1,1-dichloroethylene) as electron acceptors (Yang et al., 2017b). 16S demonstrated that a novel *Dehalogenimonas* was the most abundant organohalogen-respiring bacterium in the enrichment culture GP and named it “*Dehalogenimonas etheniformans*” strain GP. Therefore, the use and cultivation of dehalogenating respiring anaerobic bacteria is particularly important for the removal of CHCs.

### 3.2 Metabolic process of dehalogenated respiratory bacteria

**Figure 3:**
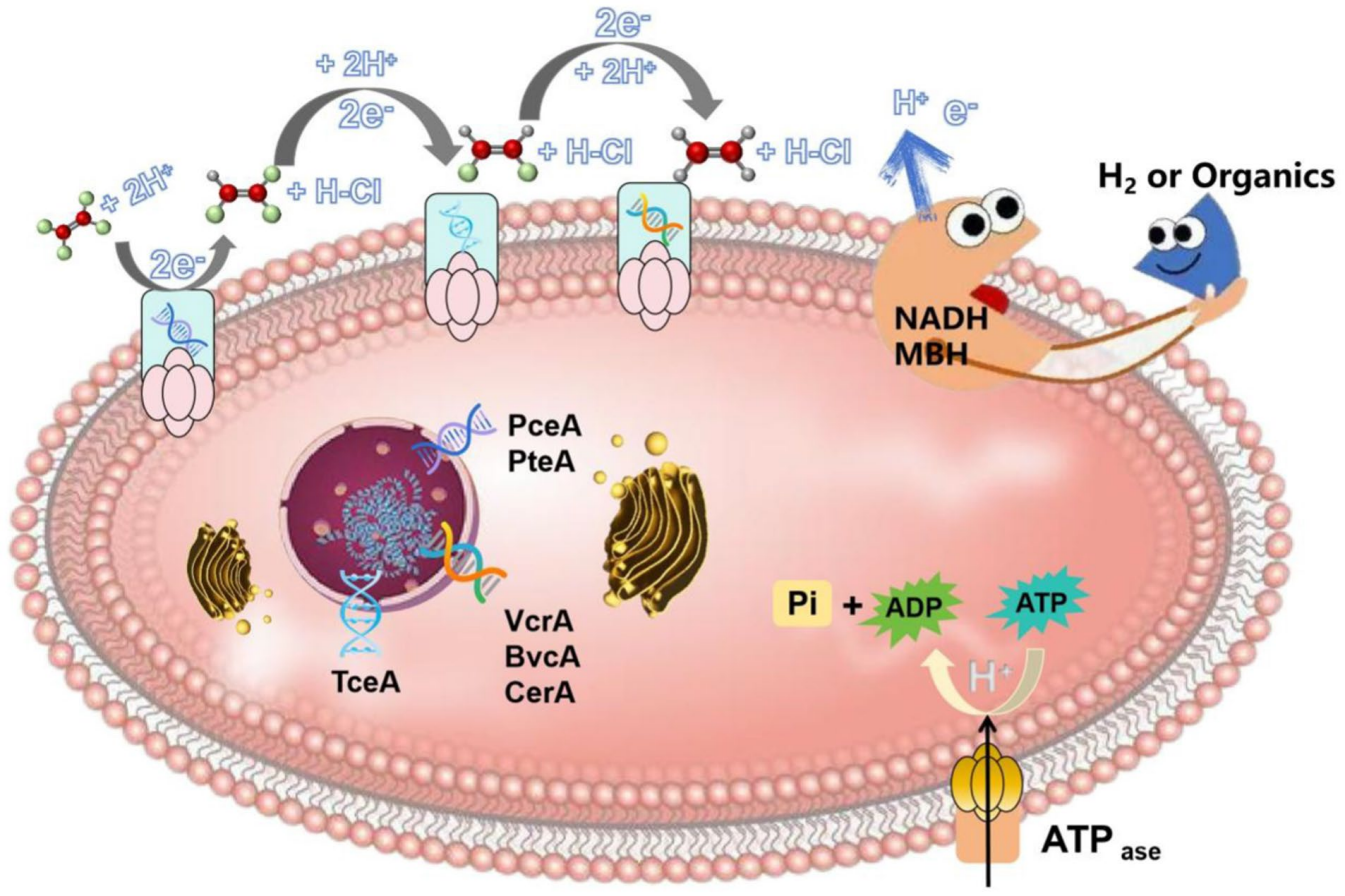
Degradation mechanism of CHCs (using perchloroethylene as an example– perchloroethylene is representative of a typical low molecular weight and widely used CHCs. In addition, the reductive dechlorination process involves multiple reaction steps, which can fully demonstrate the complexity and diversity of CHCs removal by dehalogenating bacteria and help to understand the dechlorination mechanism in depth).

The process of microbial reductive dechlorination is usually carried out by dehalogenating respiratory bacteria. The schematic diagram of its degradation mechanism is shown in Figure 3, dehalorespiratory anaerobes adsorb and contact CHCs via proteins or other biomolecules on their surfaces. And then microorganisms use endogenous electron donors such as (nicotinamide adenine dinucleotide) NADH or other coenzymes to catabolize (membrane-bound hydrogenase MBH) hydrogen or other organic compounds to produce electrons and H^+^, which attack the chlorine atoms in the CHCs molecule to reduce them to chloride ions. The essence of the reduction of CHCs is actually the transfer of electrons from the carbon-chlorine bond of CHCs to the electron transport chain of microorganisms. This process involves several electron transport proteins, coenzymes and genes (*PceA*, *PteA*, *TceA*, *VcrA*, *BvcA*, *CerA, rdhA, rdhR, dhlA*, and *mtaA*). For example, in *Dehalococcus*, *rdhA* and *rdhR* are responsible for the transcriptional reduction of the cobalamin cofactor (VB12) and the iron–sulfur cluster in the dehalogenase. The electrons generated by NADH and/or MBH are first transferred to the iron–sulfur cluster, which then transfers the electrons to the cobalamin cofactor, activating the cobalamin to a low-valent state [such as Co(I)]. The activated cobalamin reacts with CHCs to form the corresponding dechlorinated products (Agarwal et al., 2017). In *Xanthobacter autotrophicus* and *Pseudomonas*, *dhlA* is responsible for regulating dehydrohalogenase, which eliminates a chlorine atom and an adjacent hydrogen atom to form a double bond, thereby producing the corresponding olefin compounds (Ang et al., 2018). The work of dehydrohalogenase is inseparable from the support of cobalamin cofactors or iron–sulfur clusters. In the genus *Desulfovibrio*, *mtaA* encodes a methyltransferase dehalogenase that transfers a methyl group (-CH₃) to a CHCs, replacing the chlorine atom (Jugder et al., 2015). This process requires NADH to transport electrons through the electron transport chain to the active site of the dehalogenase. *Dehalogenated bacterial* electron shuttling can be achieved by direct contact or indirect mediation. In the case of direct contact, electrons can be transported directly to the CHCs via release from bacterial extracellular extramembranous vesicles or the cell surface. In the case of indirect mediation, intracellular electron shuttles are released into the external medium and react with CHCs in the extracellular space. Specifically, the use of H_2_ as an electron donor and electron phosphorylation via electron transfer from oxidation of H_2_ to reductive dechlorination of CHCs involves membrane-associated oxidoreductases. NADH and membrane-bound hydrogenase (MBH) are the initial oxidants capable of accepting electrons and H^+^ released from molecular H_2_ and/or organic substrates and play an important role in the respiratory process of *Dehalobacteria*. The process of reductive dechlorination of CHCs is usually catalyzed by a system of dehalogenases coupled to ATP synthesis, and the reductive dehalogenase enzymes (Rdases) replace the halogen substituent with a hydrogen atom, reducing toxicity and resistance to biodegradation (Maphosa et al., 2010). The process of dechlorination of CHCs releases energy, which is normally used for cellular metabolic activities of microorganisms, such as ATP synthesis, cell growth and maintenance of cell structure. The dechlorinated carbon skeleton of organic molecules can be further degraded by microorganisms and reused as a carbon or energy source (Yu et al., 2021). Most of the dehalogenating respiratory anaerobes have a limited dechlorination capacity and are only able to reduce TCE to metabolites such as vinyl chloride. Only a few *Dehalococcoides* (*Dehalococcoides mccartyi strains BTF08 and 195*) and *Dehalomonas* (*Candidatus Dehalogenimonas etheniformans strain GP*) were able to completely dechlorinate TCE and dechlorinate the metabolites to non-toxic ethylene (Kumar et al., 2015). When the toxic metabolites dichloroethylene and vinyl chloride accumulate to a certain concentration, they become the rate-limiting step of dechlorination, which is determined by the abundance of the genus *Dehalococcus*.

*Dehalococcoides* are nutritionally deprived, which results in a small genome that is inherently lacking in the ability to synthesize a wide range of growth factors. One can rely on exogenous sources to achieve optimal growth of *Dehalococcoides*; the other is obtained endogenously using complexes of nutrient exchange networks in coexisting populations (e.g., local hydrogen-producing microbial communities). In addition, *Dehalococcoides* are exposed to a wide range of environmental factors when using CHCs under more stringent growth conditions (Chen and Wu, 2022).

### 3.3 Influencing factors of dehalogenated *Pseudomonas* in treating CHCs

#### 3.3.1 The optimal survival temperature for reductive dechlorination of *dehalobacteria*

The regulation of temperature represents one of the most crucial mechanisms for influencing the metabolism of dehalogenating bacteria in an anaerobic environment. The abiotic and biological transformation of CHCs depends on various factors, including temperature, pH, carbon source, nitrogen source and oxygen concentration, all of which can either promote or inhibit the formation of electron donors and acceptors essential for dechlorination (Freitas et al., 2015). In particular, temperature impacts the intermediate reaction processes of dechlorination (such as acetic acid synthesis), which provide high quality electron donors (hydrogen) or carbon sources to facilitate the dechlorination reaction (Adrian and Löffler, 2016). Similarly, temperature fluctuation affect the lipid composition and protein conformation of microbial cell membranes, thereby influencing their permeability and stability. Higher temperatures induce the melting of cell membrane lipids, increasing permeability and facilitating the entry of substrates and the efflux of products; at lower temperatures, the cell membrane becomes stable and can affect the activity of dehalogenases and transporters (Fernandez-de-Cossio-Diaz and Vazquez, 2018). Within a specific temperature range, microorganisms will accelerate the use of intracellular reserves and promote the activity of their antioxidant system and dechlorination activity (Zhang and Wang, 2022). Therefore, the microbial-driven degradation process achieves optimal efficiency within a certain temperature range (e.g., *psychrophilic bacteria* 0–20°C *mesophilic bacteria* 8–48°C, *thermophilic bacteria* 40–70°C, and *hyperthermophilic bacteria* 65–90°C) (Omelon, 2016). Once this temperature threshold is exceeded, the life activities of microorganisms, such as protein transport and gene expression, are irreversibly damaged, leading to their death. Below this temperature, viability is compromised at the same cost.

**Figure 4:**
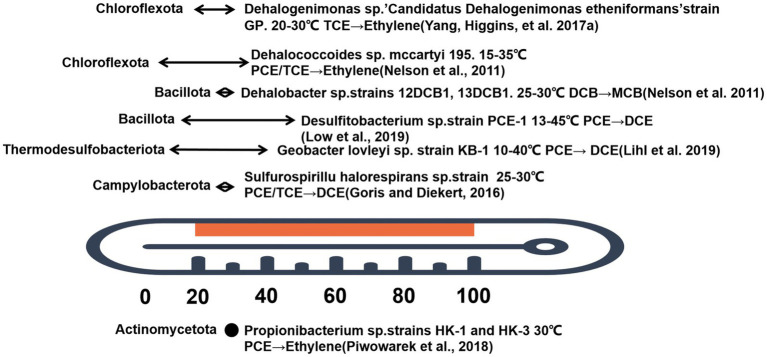
Degradation of CHCs by different bacterial genera at varying acclimating temperatures.

In general, groundwater temperature remains stable and correlates with depth, gradually increasing toward the Earth’s center (Kurylyk et al., 2019). In shallow aquifers, groundwater temperature is relatively stable, but groundwater temperature is lower than the optimal growth temperature of *Dehalobacteria*. Where human activities are present, such as underground thermal energy storage for heating and/or cooling of buildings, shallow aquifer temperatures are strongly influenced (Fleuchaus et al., 2018). By regulating the temperature of underground aquifers can increase the number of microorganisms and improve the efficiency of contaminant removal. Test results show (Němeček et al., 2018) that microbial degradation of CHCs in heated aquifers is significantly accelerated in the presence of abundant substrates. Increases in dissolved iron and sulfide in groundwater and decreases in sulfate concentrations suggest that substrate application and/or elevated temperatures provide favorable conditions for microbial dechlorination. The fastest decrease in TCE concentrations was observed in groundwater, which was most affected by heating and substrate. The following graph shows the temperature activity range of currently studied dechlorinating microorganisms (Nelson et al., 2011; Goris and Diekert, 2016; Yang et al., 2017a; Piwowarek et al., 2018; Lihl et al., 2019; Low et al., 2019; Büsing et al., 2020). At least at present, dehalobacteria are mainly distributed in several phyla (Figure 4): *Chloroflexi*, *Bacillus*, *Thermodesulfobacteria,* and *Campylobacter*. There are currently two genera that can completely dechlorinate CHCs: *Dehalococcoides* (phylum: *Chloroflexota*) and *Desulfobacterium* (phylum: *Bacillus*). Within the *phylum Chloroflexota*, three genera are active in reductive dechlorination: *Dehalogenimonas*, *Dehalococcoides,* and *Dehalobium*. The mccartyi strain grows at moderate temperatures. As shown in Figure 4, the optimal growth temperature of *mccartyi 195* is 15–35°C, and death does not occur at 40°C without dechlorination (Yang et al., 2017b). *Dehalobium chromocoercia strain DF-1* was active at 30–33°C, whereas no dechlorination occurs at 10°C or 35°C (Miller et al., 2005; Kittelmann and Friedrich, 2008). *Candidatus Dehalogenimonas etheniformans strain GP* is psychoactive at 20–30°C and can use H_2_ for reductive dechlorination of TCE and its products (Yang et al., 2017b). The optimal temperatures of other strains (as shown in Figure 4) such as *KB-1*, *HK-1*, *HK-3,* and *PCE-M2* are 10–40°C, respectively.

#### 3.3.2 Effect of pH on the microstructure of dehalobacteria and CHCs

The pH affects the ionic balance and protein structure both inside and outside microbial cells and has a direct effect on the growth and metabolic activity of microorganisms. Bioremediation of chlorinated solvent sites often results in groundwater acidification due to substrate application, fermentation and reductive dechlorination processes. The reductive dechlorination process of *Bacillus dehalogenans* is stable at neutral pH. When the groundwater pH is lower than 6.0, the microbial activity decreases rapidly and the increase in hydrogen ion concentration may damage the membrane structure and enzyme activity of the microorganisms, resulting in inhibited growth of the microorganisms. Under low pH conditions, the main microorganism responsible for dechlorination is *Thiospirillum*. The activity of cultures dechlorinating tetrachloroethylene (PCE) was not maintained in pH 5.5 media. *Sulfurospirillum multivorans* is able to dechlorinate PCE to *cis*-1,2-dichloroethylene (cDCE) in pH 5.5 media and retains this activity after repeated transfers. Inability to maintain *dehalococcal* activity is a key factor in low dechlorination rates or even cessation of dechlorination by *in situ* PCE reduction at low pH conditions (Yang et al., 2017a).

The effect of pH on the electronic structure of CHCs is mainly affects their dissociation and ionization balance. At lower pH levels, the presence of large amounts of H^+^ in solution interacts with the negative electron cloud in the CHCs molecules, causing some of the CHCs molecules to dissociate, losing their chlorine atoms and taking on a positive charge, resulting in the molecules as a whole taking on a degree of polarity. This is because an increase in H^+^ leads to a redistribution of the electron cloud in the CHCs molecule toward H^+^, potentially increasing the electron density around the chlorine atom. And under lower pH conditions, the electron donor is more likely to release electrons to stabilize its own charge, which is conducive to microorganisms carrying out dechlorination reactions (Jia et al., 2017). At high pH levels the lower concentration of hydrogen ions and more hydroxide ions in solution, which can inhibit the dissociation of CHCs, making them more likely to exist in molecular form (Liu Q. et al., 2021). At low pH levels, the partial dissociation of CHCs molecules and changes in electron cloud density make them more susceptible to interaction with electron donors in microorganisms, facilitating the dechlorination reaction. Under high pH conditions, CHCs molecules are relatively stable and their interaction with microorganisms is weakened, which is not conducive to the development of the reaction (Wang Z. et al., 2021).

#### 3.3.3 The working environment of dehalobacteria: oxygen

*Dehalobacteria* are strictly anaerobic organisms and the presence of oxygen will have some effect on their growth and degradation of contaminants. *Dehalobacteria* normally use CHCs as their main electron acceptor for respiration, thereby promoting the dechlorination and degradation of CHCs. However, oxygen is another preferred electron acceptor. In the presence of oxygen, oxygen can compete with CHCs for electrons, inhibiting the dehalogenation process. High concentrations of oxygen can cause *dehalobacteria* poisoning and loss of cell viability. Oxygen toxicity can cause *dehalobacteria* cell membrane damage, increased intracellular oxidative stress and DNA damage. In addition, under oxygen-rich conditions, *dehalobacteria* may prefer to use metabolic pathways such as aerobic respiration or fermentation rather than dechlorination pathways. In the case of short-term exposure to oxygen, cell viability can be restored after a period of time, whereas long-term exposure to oxygen will cause dehalogenating bacteria to cease dechlorination. Studies have shown (Wen et al., 2017) that dehalogenating respiratory bacteria can completely remove trichlorethylene in 5 days under anaerobic conditions, but it takes 15 days after the addition of 0.2 mg/L oxygen. The effect of oxygen on micro-organisms is irreversible.

#### 3.3.4 Plus carbon and nitrogen sources

In addition to environmental factors, dehalogenating bacteria require carbon and nitrogen sources for the dechlorination process. Most dechlorinators require acetate as a carbon source. Sodium acetate is the most commonly used carbon source in the current study and its addition significantly increases the microbial colonization rate. Studies have demonstrated that the extent of TCE reduction varies depending on the hydrogen-producing substrate, with acetate serving as a slow-releasing substrate that sustains the growth of dehalogenating bacteria over extended periods (Chen and Wu, 2022). When the carbon source is low and the concentration of CHCs is high (Cupples et al., 2004), dehalogenating bacteria may face a nutrient-poor situation, leading to a decrease in dechlorinating enzyme activity. Conversely, under conditions of high carbon sources and low CHC concentrations, these bacteria may divert to non-dechlorinating metabolic pathways to utilize excess carbon sources. This shift can lead to the production of toxic acid byproducts instead of dechlorination. Moreover, excessive carbon sources can result in overpopulation of dehalogenating bacteria, surpassing the environment’s carrying capacity, leading to resource competition and accumulation of toxic substances. Additionally, carbon sources possess detoxifying properties that aid dehalogenating bacteria in combating toxins and enhancing their adaptability to harsh environments. For example, organic acids such as acetate and propionate, water-soluble carbon sources such as glucose (Chen et al., 2018), and woody wastes such as lignin or cellulose containing lignin (Yu et al., 2023) can provide additional energy due to their ease of metabolism and utilization within the biological system, thus enhancing the antitoxicity of dehalogenating bacteria.

Nitrogen sources are important components of biomolecules such as proteins and nucleic acids. An appropriate amount of nitrogen source can promote the synthesis and repair of cell membranes, maintain the balance of osmotic pressure inside and outside the cell, and protect the internal structure of the cell from damage by the external environment (Chen et al., 2013). The cell wall of dehalogenated bacteria is mainly composed of peptidoglycan and peptidoglycan inositol, and the supply of a nitrogen source affects the synthesis and degradation process of these polysaccharides, which in turn are involved in cell wall synthesis and the maintenance of structural protein stability (Yu et al., 2023). Common nitrogen sources include ammoniacal nitrogen (e.g., urea and ammonium salt), nitric nitrogen (e.g., nitrate) and organic nitrogen (e.g., protein and amino acid) (Bin Hudari et al., 2022). While ammonium salt is a commonly used nitrogen source, it has been shown (Kaya et al., 2019) that the addition of NH_4_^+^ produces *cis*-1,2-dichloroethene (cDCE), the dechlorination rate can be increased about 5-fold, and the copy number of the 16S rRNA gene of *Dhc* incubated with NH_4_^+^ was about 43-fold higher than that of microorganisms incubated without NH_4_^+^. In TC cultures, NH_4_^+^ also stimulated the dechlorination of cDCE to ethylene and the growth of *Dhc*.

#### 3.3.5 Other substances

There are many other substances that can promote or inhibit the growth of micro-organisms (e.g., trace elements, vitamins, substrates, etc.). Studies have shown that substrates vary in their ability to stimulate the growth and activity of dehalorespiratory bacteria. For example, dichloroethylene can inhibit the vital activity of *Dehalococcoides* and even cause the strain to die, so at high TCE loads, large accumulations of dichloroethylene may exacerbate substrate effects (Delgado et al., 2014).

Moreover, studies have shown that substrate availability does not necessarily dictate the metabolic capabilities of *Dehalococcoides*. Even in the presence of external cobalamin, the growth of *Dehalococcoides* can be sustained at optimal levels (108 copies/ml). Recent research highlights that adding exogenous cobalamin at concentrations ranging from 8.1 to 34 pg./L fulfills the essential metabolic requirements of 106 *Dehalococcoides* per liter (Yan et al., 2021).

**Figure 5:**
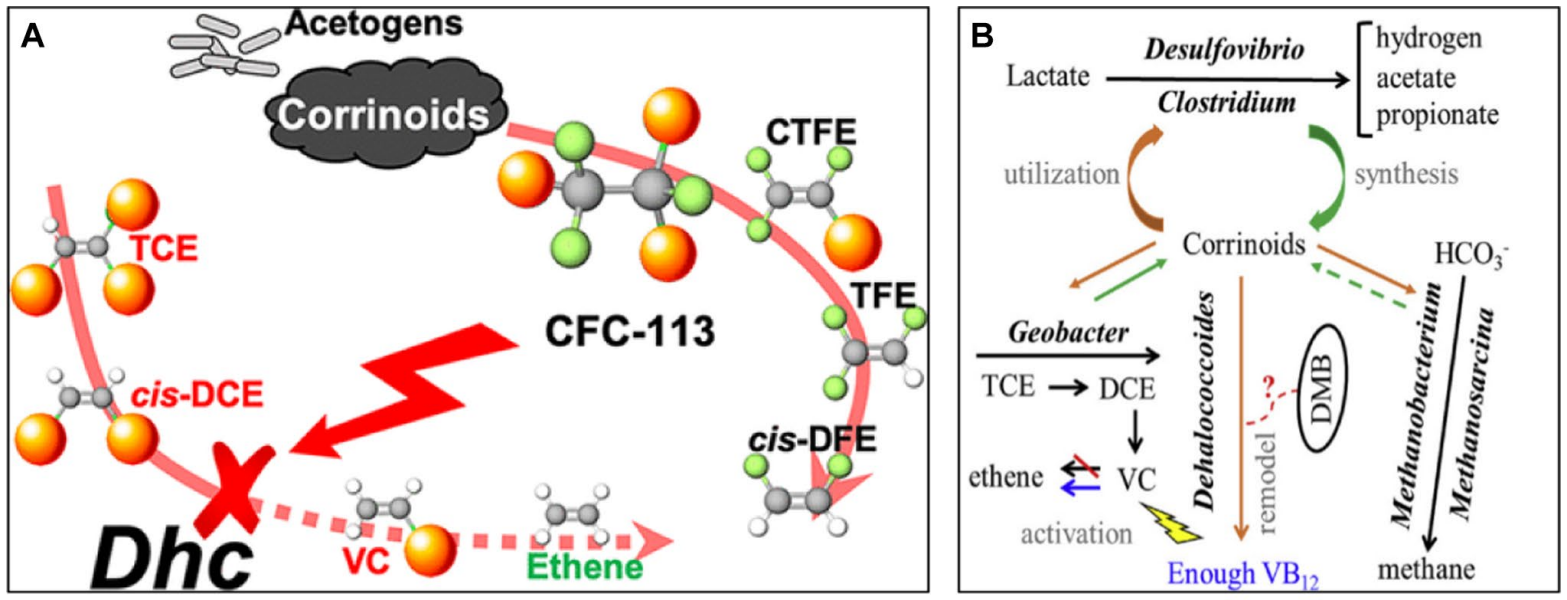
**(A)** Schematic representation of the effect of CFC-113 on trichloroethylene dechlorination. **(B)** Schematic representation of the effect of Vitamin B12 (VB12) on microbial dechlorination (Im et al., 2019; Wen et al., 2020).

Co-existing contaminants at contaminated sites may also inhibit the growth of *dehalococcoides*. Underground aquifers contaminated with TCE often contain 1,1,2-trichloro-1,2,2-trifluoroethane (CFC-113). As shown in Figure 5A, CFC-113 inhibits the reductive dechlorination activity of *Dehalococcoides mccartyi* in a concentration-dependent manner, while its degradation intermediates, CTFE, TFE, and cis-DFE, do not inhibit *Dhc* from dechlorinating TCE (Im et al., 2019).

Vitamins can also influence microbial growth. For example, VB12 restriction changes the microbial community structure of the microbiota in the manner shown in Figure 5B. In cultures without VB12, the relative abundance of *tceA* or *vcrA* in *dehalococci* was low. After three consecutive cycles of VB12 starvation, the frequency of *dehalococci* decreased from 42.9 to 13.5% (Wen et al., 2020).

#### 3.3.6 Other microorganisms

The community of dehalogenating microorganisms typically–includes fermentative and methanogenic species such as *Bacillus methanogenes*, *Octococcus methanogenes*, *Vibrio desulfuricans*, *Acetobacter,* and *Clostridium perfringens* (Hug et al., 2012; Jacome et al., 2019). The symbiotic and competitive relationships among microbial communities shift constantly as environmental conditions fluctuate. When lactate serves as the sole carbon source, lactic acid-fermenting desulphurising *Vibrio vulnificus* and *hydrogenotrophic methanogens* emerge as predominant non-desulfurizing flora, forming a stable microbial community alongside dehalogenating bacteria (Li X. K. et al., 2022). Reduced metabolites (e.g., hydrogen, lactic acid, etc.) produced by lactic acid-fermenting desulphurising *Vibrio vulnificus* and *hydrogenotrophic methanogens* during their metabolism can act as electron acceptors for dehalogenating bacteria, providing an energy sharing cycle and facilitating the reduction reaction of CHCs by dehalogenating bacteria.

There may be competition between different microorganisms for electron donors or nutrients. Research (Chen and Wu, 2022) indicates that some electrons can flow to *methanogens* under conditions where the electrons provided are sufficient for the reductive dechlorination of trichloroethane to ethylene. The growth of hydrogenotrophic methanogenic bacteria is stimulated by using sugar-oil mixtures as electron donors, and the performance of these methanogens in competition with *Dehalobacteria* varies with their fermentation kinetics (Swezy, 2016). Furthermore, under conditions of sulfate reduction, methane production, and co-acetate production, dechlorinating microorganisms compete with other anaerobic hydrogenotrophic microorganisms for hydrogen utilization. The competitiveness of these microorganisms decreases in the presence of more oxidative terminal electron acceptors (Wei et al., 2016).

## 4 Synergistic microbial treatment of biochar and its composites

The effect of biochar and its composite materials on microorganisms is twofold. On the one hand, when biochar and its composites are used in excessive amounts, they can be toxic to microorganisms and lead to their death; On the other hand, modest additions of materials may also stimulate microbial growth and dechlorination processes. A study (Semerad et al., 2021) showed that the toxicity of the material to microorganisms increased temporarily after adding 0.7 g/L of iron-carbon material, after a period of time, the stimulating effect of the material on microorganisms may exceed the negative effect, and can even change the restoration process. This nano-bio-combination remediation method combines the positive characteristics of the original agent and the growth characteristics of the microorganisms, which is a promising technological method for groundwater remediation applications.

### 4.1 The effect and influencing factors of biochar and its composite materials in synergy with microorganisms to remediate CHCs pollution

Synergistically, biochar and its composites can stimulate the growth and dechlorination processes of microorganisms, some can even regenerate and recycle biochar and its composites (Su et al., 2023). High concentrations of CHCs are toxic to micro-organisms. The adsorption and degradation capabilities of biochar and its composites provide a conducive growth environment for microorganisms, promoting the bioconversion of CHCs. A study on the degradation of 1,2-dichloroethane by microorganisms associated with biochar (Hasanan et al., 2024) found that an increase in the abundance of bacterial communities was observed after the addition of biochar, with the presence of *Bacillus* (from 1. 04 to 2.3%), *Bacteroidetes* (from 16.5 to 20.5%), *Clostridium* (from 3.4 to 5.7%), *Cyanobacteria* (from 0.07 to 5.6%), *Spirillum* (from 0.9 to 1.8%), and *Micrococcus wartyi* (from 2.9 to 3.5%). Dechlorination was tripled compared to no biochar addition (32.2 ± 6.9% to 60.2 ± 11.5%), and the addition of biochar was characterized as increasing hydrogenase activity by approximately 52%. Currently, the most commonly used biochar composites for the treatment of CHCs are iron-carbon composites. Biochar as a material substrate can effectively reduce the adverse reactions (e.g., non-specific reactions, toxicity, etc.) of nano-zero-valent iron and improve the performance of nano-zero-valent iron for reductive dechlorination (Tan et al., 2016). Zero-valent iron nanoparticles and biochar can form “electron shuttling bridges” to enhance the catalytic effect of surface functional groups involved in electron shuttling, thereby improving the dechlorination of chlorinated hydrocarbons and also help to improve the stability and mobility of nano-zero-valent iron in groundwater (Yan et al., 2015). A study on biochar-mediated activation of NZVI by *Shewa bacteria* to enhance dechlorination of pentachlorophenol (PCP) (Li et al., 2019) showed that the addition of biochar reduced the agglomeration of NZVI, increased the concentration of atomic hydrogen H* and accelerated the formation of Fe(II). Secondly, the electron shuttling property of biochar enhanced the activation of aged NZVI by *Shewa bacteria*, resulting in the transformation of aged NZVI (without biochar addition) and aged NZVI-BC600 (with biochar addition) into green rust and purple iron ore, respectively. This accelerated the reductive dechlorination of PCP and the maximum PCP degradation rate (*k*_max_) was 2.45 mg·L^−1^·d^−1^, which was 2.6 times higher than the control without biochar (0.94 mg·L^−1^·d^−1^). Biochar has a larger specific surface area, which can provide more electron transfer sites for microorganisms. Studies have shown that biochar can support microbial transformation through both the reversible redox reaction of its quinone groups and the electron conduction of its graphene domain (Saquing et al., 2016). Biochar can be both an electron donor and acceptor. As it has a considerable bioavailable electron storage capacity ESC, biochar is considered to be a rechargeable storage bank of bioavailable electrons (Xu et al., 2013). In recent years, it has been proposed that biochar can facilitate direct interspecies electron transfer (IET) by conducting electrons between microorganisms. To demonstrate that the stimulation of IET was observed in co-cultures with *Geobacillus sulphurus* or *Octococcus* bacillus despite the use of poorly conductive biochar, mainly due to the electronic conduction of biochar (Rotaru et al., 2014). By coupling biochar with microorganisms, builds electron transport tunnels and uses its own growth metabolism to hydrogenate CHCs, thereby removing it. Modified materials can better handle contaminants and are easy to separate and recycle, thus avoiding secondary environmental damage (Ai et al., 2022). A study (Wang et al., 2022) found that the initial treatment of contaminants using biochar-modified materials in cooperation with microorganisms achieved 97.9% removal of trichloroethylene from the effluent after 2.5 h, and up to 67.3% removal after seven cycles, which is 21.7% higher than that achieved by treating trichloroethylene with biochar and its composites alone.

The removal of CHCs by biochar and its composites coupled with microorganisms is the result of a combination of bioremediation and a bioremediation. The effects on the removal of CHCs are then summarized in terms of influence of nutrient substrates on the metabolism of dehalogenating bacteria in biochar mixtures, the dosage of biochar composites and the concentration of CHCs and how biochar coupled microorganisms build an “electron shuttle bridge” to remove CHCs.

#### 4.1.1 Influence of nutritional substrates on the metabolism and dechlorination of dehalogenating bacteria in biochar mixtures

Organic substrates affect microbial growth and dechlorination. It was found that some dehalogenating genera (e.g., *Dehalobacter* spp., *Shewanella* spp., *Geobacter* spp., etc.) prefer to metabolize in nZVI-rich environments and reductively dechlorinate using electrons. The addition of substrates not only affects natural organic halide respiring genera, but also increases the number of iron/sulfur reducing bacteria (Pasinszki and Krebsz, 2020). The carbon source acts as an organic substrate that promotes microbial growth and stimulates the microbial dechlorination process. Modification of slow-release carbon sources on the surface of biochar-based materials can well achieve the effect of providing carbon sources for the microbial dechlorination process. Common sustained-release carbon sources include polylactic acid, polycaprolactone, polyhydroxyalkanoates, etc. (Cui et al., 2022). Studies have shown (Ji et al., 2019) that biochar-based composites modified with polylactic acid have a better carbon source in water. The slow release effect is good and the slow release carbon source is fully utilized by the microorganisms that are stimulated in the process to promote better dechlorination. In this study, the addition of the slow-release carbon source played an important role in the microbial dechlorination process, proving the long-term effectiveness of the composites. Meanwhile, the addition of lactic acid during the degradation of trichloroethylene by the iron-carbon composites resulted in the formation of a large amount of dichloroethylene, which proved that biotransformation was involved in the removal process and the addition of whey stimulated the biodegradation process. One week after the addition of whey, complete mineralization of trichloroethylene to CO_2_ and H_2_O was achieved. The addition of substrate not only stimulates the biotransformation process, but also changes the microbial species and abundance, favoring the growth and enrichment of dehalogenating bacteria (Pasinszki and Krebsz, 2020). In the current research, there are many studies on carbon sources that can promote microbial growth, and the types of carbon sources have been extensively studied. However, there are few studies on the effects of adding organic substrates on nZVI/BC materials and resident microorganisms and this aspect needs to be further investigated.

#### 4.1.2 Effect of dosage of microorganisms coupled to biochar composites and concentration of CHCs on the removal of CHCs

Appropriate dosage of biochar composites can increase the adsorption of CHCs reducing their concentration in the water column. Coupling this with the right amount of dehalogenating bacteria can accelerate the degradation rate of CHCs, effectively reducing their residual levels. However, it should be noted that too high a dose of biochar composites can have a toxic effect on dehalogenating bacteria, limiting their growth and activity. Studies have shown that as the dose of zero-valent iron increased, the removal efficiency of the coupling system on TCE increased accordingly and correspondingly. However, when the dose exceeded 1 g/L, a negative effect was produced and the unique intermediate products of biodechlorination, dichloroethylene and vinyl chloride, were not detected during the removal of TCE and the biological process was completely inhibited and the zero-valent iron produced a toxic effect on the dehalogenating bacteria. In contrast, there was no inactivation of dehalogenating bacteria with increasing doses of biochar, which improved the microbial environment and enhanced metabolic behavior, leading to a consistently positive correlation with TCE removal (Ma et al., 2023). Too high a dose of dehalogenating bacteria may compete for resources (pores, nutrients, surfactants, etc.) on the biochar and its composites, reducing removal efficiency, while too low a dose may not fully utilize the degrading capacity of the dehalogenating bacteria, resulting in poor removal. A study titled Polycaprolactone-modified biochar-supported nanoscale zero-valent iron coupled with *Shewanella putrefaciens* CN32 for simulated removal of 1,1,1-trichloroethane from groundwater showed that 1,1,1-Trichloroethane removal rate increases with the increase of the dosage of composite materials and *Shewanella putrefactive bacteria CN32*. However, when the compound addition amount exceeds 1:100, the increase in removal rate slows down; In addition, when the dosage ratio of composite materials and *Shewanella putrefactive bacteria CN32* is 1:100, as the concentration gradient of 1,1,1-trichloroethane increases (25, 50, 100, and 200 mg/L), the degradation rate gradient decreases, and the degradation effect also gradually decreases (50 h, 90–38%). The reason for this is that 1,1,1-trichloroethane reacts strongly with nZVI when the initial concentration is relatively high, and a dense passivation layer consisting of Fe(III) oxides/hydroxides is rapidly formed on the BC surface, which hinders the reaction (Ye et al., 2023).

#### 4.1.3 How do biochar-coupled microorganisms build an “electron shuttle bridge” to remove CHCs?

The ESC is an important property that determines the ability of biochar to store and reversibly exchange electrons with the surrounding environment. With high ESC, biochar can serve as an effective medium to support abiotic and microbial reductive dechlorination. Generally, the ESC and electron exchange capacity (EEC) of biochar can be estimated and optimized by considering electrode morphology and surface functional groups. A higher specific surface area and rich microporous structure usually enhance capacitance and the charge–discharge rate. The pore size of biochar varies greatly, but the smaller pores are typically on the nanometer scale, which is the typical thickness of the electrical bilayer (Prévoteau et al., 2016). Surface functional groups (such as -OH, -COOH, and ether bonds) can adsorb ions in solution and provide additional charge transfer pathways. Higher electronic conductivity promotes rapid electron transport and electrochemical reactions. Pyrolysis is a process that produces ESC and EEC, which are common properties of biochar produced from all lignocellulosic biomass (Xin et al., 2021). Woody biochars can typically donate an order of magnitude more electrons than they can accept, facilitating reductive dechlorination reactions in the subsurface (Yuan et al., 2022). Studies have shown that biochar coupled with *Geobacter sulfurreducens* accelerates the transfer of electrons from cells to pentachlorophenol, enhancing the system’s reductive dechlorination behavior. Compared to the single system, the EEC in the coupled system is up to 24 times higher, with the electron shuttle bridge contributing to 41% of the biodegradation rate. This demonstrates that the formation of an “electron shuttle bridge” is a prerequisite for the successful and efficient reductive dechlorination of biochar coupled with dehalogenating bacteria (Yu et al., 2016).

Precise removal of CHCs can be achieved by coupling biochar and its composites with dehalogenating bacteria. Biochar provides a habitat for the growth of dehalogenating bacteria, and the rich microporous structure and stable graphitised layer provide both nutrient adsorption and some electron storage capacity to promote the degradation activity of dehalogenating bacteria.

### 4.2 Reaction mechanism

Biochar and its composites have a highly developed pore structure that adsorbs and enriches organic substrates, nutrients and trace elements from the environment, which can be used as a partial nutrient source for dehalogenating bacteria to promote their growth and metabolic activity. A study entitled Biochar (BC) and Biochar-Polylactic Acid Composite (PBC) Enhancement of Hexachlorobenzene Biodegradation in Soil (Acrisols) by Alteration of Microbial Community showed that for acrisols, the evenness, Simpson’s diversity and effective species of the PBC treatments were higher than those of the CK and BC treatments by 0. 2–0.3%, 3.3–3.4% and 39.6–42.1% (*p* < 0.05); and the number of *Azotobacter* spp. and *Pseudomonas* spp. belonging to the phylum Aspergillus were significantly higher in comparison with the CK and BC treatments; meanwhile, the functional characteristics of “carbohydrate metabolism,” “amino acid metabolism,” “cofactor and vitamin metabolism,” “lipid metabolism,” “signal transduction,” “xenobiotic degradation and metabolism” and “transport and sorting degradation” were higher in PBC than in BC than in the CK and BC treatments in the same soils. Characteristics were more common in PBC than in BC and CK (p < 0.05) (Fan et al., 2022). Biochar and its composites act as pH buffers in the water column to maintain a suitable environment for the growth of dehalogenating bacteria and the modified biochar has higher electrochemical activity and richer reactive groups than the precursor, which influences and facilitates the electron transfer process and anaerobic respiratory energy metabolism process of dehalogenating bacteria (Song et al., 2024).

**Figure 6:**
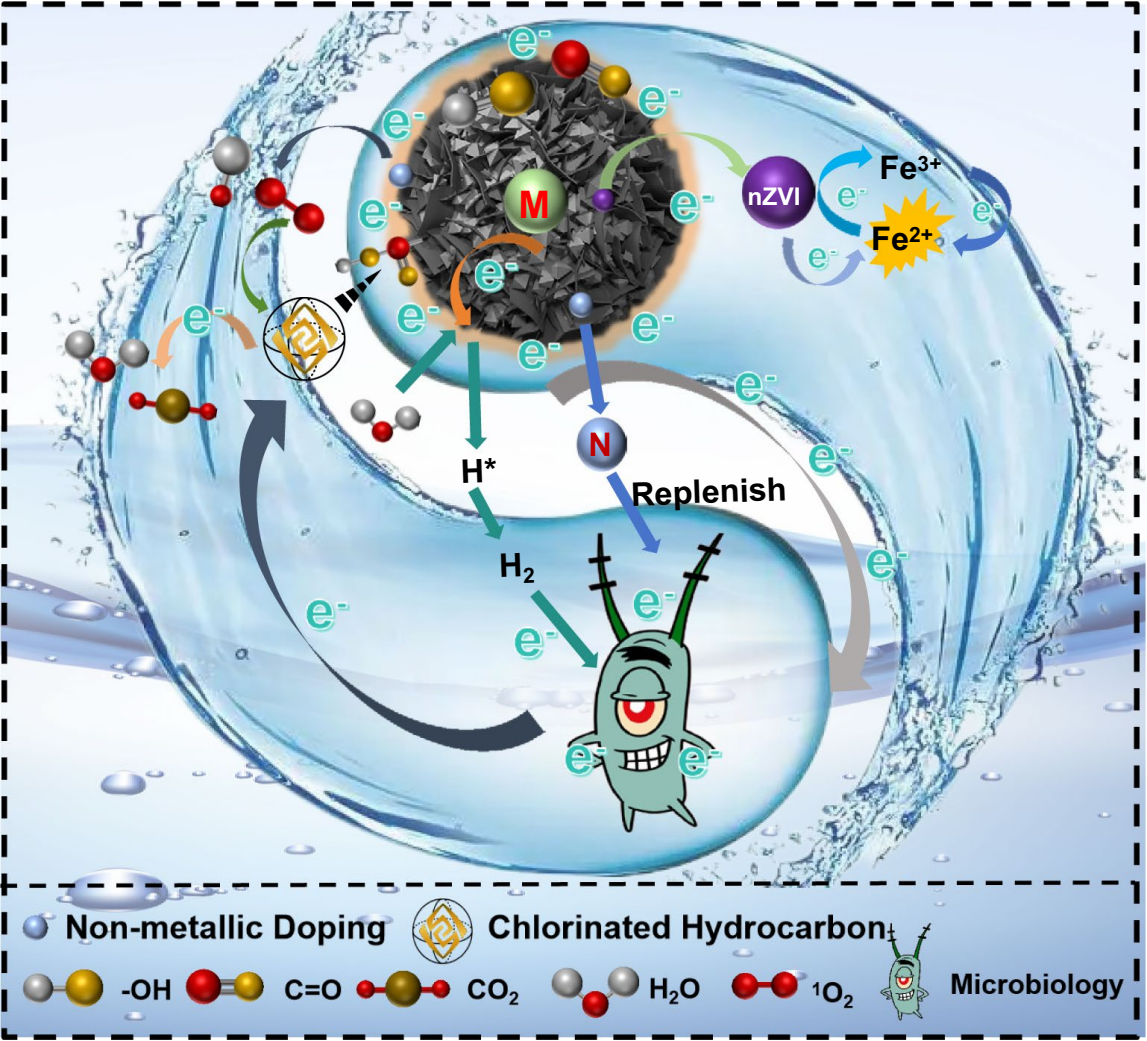
Mechanism of coupled microbial removal of CHCs by biochar and its composites.

The process of CHCs removal by biochar and its composites, dehalogenating anaerobic respiratory bacteria and their synergistic effect is shown in Figure 6. In the coupling system of biochar and its composites with dehalogenating bacteria, dehalogenating bacteria use “electron bridge” (such as biochar with stable graphitised structure) to accept exogenous electrons generated by biochar composites or use metabolic substrates to generate electrons, which in the process of passing through the electron transfer chain, generate proton gradient and Driving intracellular ATP synthesis to sustain the survival and metabolic needs of the dehalogenating bacteria, thus enabling the dechlorination process (Tang et al., 2022). The addition of biochar and its composites not only provides a good habitat for dehalobacteria, but also plays a key and enhancing role in the electron shuttle process: (I) after biochar and its composite materials release electrons through their own active substances, the electrons act on the dehalogenating bacteria through the “electron bridge” constructed by the biochar and its composite materials, and are used for reductive dechlorination after activating the dehalogenating bacteria; (II) dehalogenating bacteria produce electrons when metabolizing substrates, and these electrons are transported through the conductive structure of the biochar and its composites (Patwardhan et al., 2022). Electrons are transferred through conductive channels in biochar and its composite particles and ultimately act in the reduction process of CHCs. It has been shown (Meng et al., 2018) that the treatment of CE by nZVI/BC composites is a stepwise dechlorination process. As can be seen from the transformation products, the transformation process involves both biotic and abiotic transformation mechanisms. When nZVI was completely depleted, acetylene was detected, suggesting that dissolved iron produced by bioreduction of insoluble iron minerals was involved in the reductive DCE dechlorination. In addition, their presence was associated with further removal of dichloroethene and vinyl chloride, as well as the formation of non-chlorinated volatiles, including acetylene. Meanwhile (Ji et al., 2019), modification of biochar with surfactants such as dopamine can introduce new oxygenated functional groups, which can help to improve the electrochemical activity of the material, accelerate the chemical reaction rate and also stimulate microorganisms to carry out the dechlorination reaction.

## 5 Conclusion and prospects

This paper summarizes the literature on biochar and its composites, dehalogenating respiratory anaerobes and synergistic treatment of CHCs in water with biochar and its composites and microorganisms. The following conclusions can be drawn from the review:

1. Biochar removes CHCs mainly by relying on its own unique physicochemical properties (e.g., chemical bonds, functional groups, adsorption sites, etc.); Modified biochar produced by doping with non-metallic and metallic elements, or by injecting electron-generating reactive substances directly into the reaction system, rely on the generation of atomic hydrogen H*, persistent free radicals and electron shuttling to reduce CHCs, resulting in true dechlorination, which is more than a simple adsorption process.
2. Process for the treatment of CHCs in water by dechlorination using respiring anaerobic bacteria. The appropriate temperature range for metabolic dechlorination is generally no higher than 25–30°C and the appropriate pH is 7–8. It must be carried out under strictly anaerobic conditions. Since dehalogenating bacteria are auxotrophic and have a small genome, they must add an external carbon source or obtain it endogenously by using the complex nutrient exchange network in the coexisting population. The nitrogen source stimulates microbial growth and metabolism and accelerates dechlorination. In addition, the regulation of dehalogenase is inseparable from the synthesis of cobalamin cofactors and iron–sulfur clusters, so an appropriate amount of exogenous VB12 must be added to support synthesis. The basic mechanism of dechlorination is that surface proteins or other biomacromolecules are adsorbed and brought into contact with CHCs. The microorganisms then use endogenous electron donors such as NADH or MBH to decompose hydrogen or other organic compounds to produce electrons and H^+^ which attack the chlorine atoms in the chlorinated hydrocarbon molecules, reducing them to chloride ions.
3. The reduction of CHCs by biochar and its composites, coupled with dechlorinating bacteria, is essentially an electron shuttling process. The dehalogenating bacteria use biochar and its composites to construct an “electron shuttle bridge” to accept exogenous electrons generated by the bacteria or to use metabolic substrates to generate electrons. As these electrons pass through the electron transport chain, they create a proton gradient that drives intracellular ATP synthesis. This ATP synthesis sustains the dehalogenating bacteria’s survival and metabolic needs, thereby enabling the dechlorination process.

More relevant research is needed in future studies:

1. The addition of nitrogen sources, carbon sources, vitamins, amino acids and other substances will promote or inhibit the growth of micro-organisms to varying degrees. However, very few studies have demonstrated the effects of these added substances on groundwater and other organisms in the surrounding environment. In actual projects, it is crucial to pay attention to the impact of these substances on the groundwater system and the surrounding environment to avoid secondary pollution.
2. There are currently fewer studies on the use of biochar and its composites with microorganisms to treat CHCs in groundwater. Secondly, most of the raw materials for biochar come from wood, sewage sludge, fecal matter and so on. We can look further afield and expand the range of materials to include seafood such as fish, shrimp and shellfish, which are rich in trace elements and minerals that are conducive to synergistic microorganisms at a later stage. The synergistic effect can improve treatment efficiency. The process may involve biostimulation and material regeneration and further research is needed. Systematic studies of the kinetics, microbial adaptations and synergistic effects throughout the dechlorination process remain critical. In the future, this information will shed light on bioremediation, dechlorination and the chemical industry through biotechnological innovation.

## Author contributions

SN: Data curation, Methodology, Writing – original draft. CL: Funding acquisition, Software, Writing – original draft, Writing – review & editing. SG: Data curation, Funding acquisition, Methodology, Writing – original draft. JT: Data curation, Methodology, Writing – original draft. CZ: Investigation, Methodology, Writing – original draft. LL: Formal analysis, Writing – original draft. YH: Conceptualization, Funding acquisition, Writing – original draft, Writing – review & editing. HL: Conceptualization, Funding acquisition, Writing – review & editing.
